# Economic Benefits of Diagnostic Testing in Livestock: Anaplasmosis in Cattle

**DOI:** 10.3389/fvets.2021.626420

**Published:** 2021-08-03

**Authors:** Ashley F. Railey, Thomas L. Marsh

**Affiliations:** ^1^Department of Sociology, Indiana University, Bloomington, IN, United States; ^2^School of Economic Sciences and Paul G. Allen School for Global Animal Health, Washington State University, Pullman, WA, United States

**Keywords:** diagnostic testing, anaplasmosis, economics of animal disease, transboundary, United States

## Abstract

Anaplasmosis is a costly livestock disease that persists across the United States and the world. While the traditional control options of feed additives, vaccination, and post-infection antibiotic treatments exist, the highly infectious, often asymptomatic onset of anaplasmosis in cattle makes the optimal combination of disease control measures uncertain. Reducing the infection uncertainty through early detection may help producer management decisions and reduce the economic impact of anaplasmosis. To address this, we calculate the costs of applying a range of anaplasmosis control decisions for a representative cow-calf producer in the United States and extend existing analyses to incorporate early detection through diagnostic testing. We use parameters from extant literature, including for mortality, morbidity, and treatment costs to populate a stochastic, dynamic model. Updating the cost estimates finds that production losses account for the majority of anaplasmosis costs, following previous empirical estimates. Using these estimates in our decision model, the outcomes suggest that diagnostic testing with preventative treatments is the optimal herd management strategy. By further framing our findings in the context of three anaplasmosis infection regions in the United States (endemic, disease free, non-endemic buffer), we show that additional considerations exist, which can make sub-optimal control strategies competitive. Our analysis provides an initial exploration of the economic feasibility of diagnostic testing, while helping to assess the burden of anaplasmosis more accurately.

## Introduction

The production losses and costs to treat anaplasmosis impose significant economic burdens on cattle sectors worldwide (1–3). The emergence of anaplasmosis in a herd can cause a 30% increase in the cull rate, 20–30% loss in body weight, and death or abortions in clinically infected animals (4). In the United States, death loss and treatments of infected animals on average account for the majority of total disease costs (5).

A producer's ability to minimize anaplasmosis treatment costs and productivity losses in the United States is hampered by infection uncertainty from late detection of infected animals and geographic variation in seroprevalence. Infection, through the transfer of the blood pathogen, *Anaplasma marginale*, occurs quickly, often without symptoms, and with high infection rates amongst susceptible populations. In disease-free areas, late treatment can further increase the infection rate such that culling to reduce transmission becomes the optimal strategy (6). Cattle that survive infection gain immunity but become carriers for mechanical and tick-borne transmission to spread infection within the herd and sustain the disease within a region (7, 8). Immunity in adult cattle historically corresponds geographically and seasonally with warm, wet climates where the disease is considered endemic (9). Increasingly, though, the widespread movement of animals and changing climates have contributed to wider incidence in previously disease-free areas (10, 11). In these “non-endemic buffer regions” with unknown seroprevalence, the potential economic impact of infection is high as cattle have likely not been previously exposed to anaplasmosis.

Early detection to identify the presence of anaplasmosis either within a herd or amongst animals introduced to a herd represents a pivotal decision point for the producer to influence production losses and, subsequently, cost outcomes. To capture a producer's decision uncertainty in identifying anaplasmosis, we incorporate early detection through diagnostic testing into common disease management strategies for a representative United States cow-calf producer across a range of herd infection and seropositivity levels. The commercially available enzyme-linked immunosorbent assay (cELISA) is timely and accurate (12–15), making it the preferred serological test for identifying anaplasmosis by regulatory authorities and for movement in and out of endemic regions (16, 17). Use of the test may equally be beneficial in disease-free regions by detecting infected animals with sufficient time to enact disease control measures and reduce losses (18), while also distinguishing seropositive animals to reduce unnecessary treatment (19). The costs of existing, commonly used anaplasmosis controls through preventative chlortetracycline feed additives and vaccination, as well as post-infection treatment with antibiotic injections have previously been estimated (4, 5, 20), but no cost estimates exist for diagnostic testing as part of anaplasmosis control strategies.

To incorporate diagnostic testing into an individual producer's anaplasmosis herd management strategy, we assume producers face an exogenous probability of infection and assess the cost tradeoffs between early detection with treatment or prevention of anaplasmosis, compared to production losses and costs. We use a stochastic, dynamic decision model that includes infection uncertainty, base, herd level anaplasmosis seropositivity, early detection through testing, and economic losses across decision points. We collect the cost estimates for anaplasmosis losses and treatment from local market prices, expert opinion, and by adjusting historic estimates for inflation. Empirical ranges in production losses and costs introduce uncertainty into the cost estimates. Unlike existing studies that focus on the statistical details of a diagnostic test (19), or the epidemiological modeling of anaplasmosis infection (6), the decision model occurs across disease states and test outcomes to focus the economic decision process of an individual producer on diagnostic testing and its impact on a producers behavior to optimize revenues and costs. Thus, our analysis serves to guide producers with limited disease information on minimizing production losses, given a range of disease management scenarios. Our analysis does not necessarily provide a definitive cost benefit analysis of anaplasmosis, nor is it a comprehensive model of the economic and epidemiological outcomes of anaplasmosis.

## Materials and Methods

### Economic Decision Model

Our analysis follows a profit maximizing producer, with limited disease information, who implicitly minimizes costs over a set time horizon. Anaplasmosis occurs seasonally, with progression from infection to a clinical case occurring in 3–8 weeks (11). The producer's control strategy includes either applying low doses of chlortetracycline feed additives over several months during the height of biological spread (21), administering vaccines with boosters every year to 2 years to induce immunity (9, 22), or applying post-infection therapeutic antibiotic injections to abate the severity of infection (21). The finite infection timeline and control strategies restrict the producer's decision to two production seasons and excludes considerations for optimal control strategies over an infinite time horizon or incorporating uncertainty in revenue from potential market fluctuations. Subsequently, the producer considers opportunity costs of losses across the two seasons. The producer choses a control strategy for the entire herd, instead of animal by animal, but chooses disease control efforts based on breeding cows and bulls as these animals are important assets and incur substantial economic impacts (5). Diagnostic testing increases the cost per animal treated but may lower total production costs by preventing future losses. The savings in cost from testing can include fewer future treatments of sick animals, as well as less labor and management resources needed for herd health activities in the long run.

### Production Costs and Losses

To establish production costs and losses we follow two approaches. We first calculate the expected costs of anaplasmosis infection by relying on existing, empirical estimates and adjusting to 2016 values, in USD, with the Producer Price Index (Table 1) (4, 5, 20, 24). Based on the price changes from 1980 to 2016 in the Producer Price Index, the annual inflation rate was roughly 2.4%.

**Table 1:** Parameters to calculate the expected cost of a clinical case of anaplasmosis.

	**Unit**	**Parameter**	**Notes/Sources**
**Production losses**			
Weight (lbs)	Cow	1,400	Average weight of animals based on empirical studies (5, 20)
	Bull	2,000	
Chronic Case	Cow	900	Average weight loss. Considered not to regain weight (5, 20)
	Bull	1,250	Weight of replacement bull (5, 20)
Abortion (lbs)	Heifer	500	Average weight loss of infected animals from 1980 empirical studies (20)
	Steer	550	
**Rates**			
Death Loss	Cow	0.325	Assumed death loss. Upper and lower bound ranges for sensitivity analyses are (0.30, 0.50) and (0.01, 0.05), respectively (20)
	Bull	0.035	
Weight Loss	Cow/Bull	0.210	Average proportion of weight lost in cows and bulls, based on empirical estimates (4, 20)
Chronic Case	Cow	0.250	Proportion of chronic cases in cows/bulls based on empirical estimates (5)
	Bull	0.030	
Culling Rate	Cow/Bull	0.064	Proportion of animals culled based on empirical estimates (20)
Abortion Rate		0.067	Based on 1980s number of abortions out of total heifers/steers with a 90% calving rate (5)
Treatment Rate	Cow	0.675	Treatment of remaining, uninfected cows or bulls, respectively
	Bull	0.965	
Herd Size	Cow	0.962	Proportion of clinical case in cows based on empirical estimates (4, 5, 20)
	Bull	0.038	
Market Value-Average ($/lb)	Cow	0.640	Based on average 2016 slaughter prices in the US (23)
	Bull	0.470	
Inflation Rate	Cow/Bull	0.024	Adjustment rate, based on changes in the Producer Price Index (24)
**Costs (in USD)**			
Death	Cow	1,500	Assumed current value of cow and bull.
	Bull	2,500	
Culling	Cow	285	Accounts for weight loss and lost revenue from lower market prices (5)
	Bull	1,560	
Abortion	Heifer	600	At $1.20/lb for heifer and $1.30 lb for steer. Calculation of total cost assumes 50/50 ratio of losses (4, 5, 20)
	Steer	715	
Treatment	Cow/Bull	150	Total average cost of treating entire herd, including labor costs, dispensing fees, and exams of animals.^c^
Diagnostic Testing ($/head)	Cow/Bull	7	Cost for blood draw included in range, which is reported at $0.50/cow for labor, equipment, and needles^a^
Vaccination ($/head)	Cow/Bull	11.7	Annual cost of one vaccination per animal, with labor costs, given a range of $8–15.
Pre/Post Antibiotics ($/head)	Cow/Bull	37.4	Based on 4 treatments, includes 1 h of labor for each treatment, given a cost range of $30–40.^c^
Preventative Feed ($/head)	Cow/Bull	13.8	Based on one dose per day for 120 days, with labor of 30 min per day, given a range of $10–16.^c^
Farm Labor ($/herd)	Cow/Bull	17	Farm labor rate per hour, based on Kansas rates 2015.^b^ Used to calculate parameter estimates for vaccination, antibiotics, and feed additives (25)
Discount	Herd	0.040	Applied to determine the input costs of herd vaccinations, antibiotics, feed, and other treatments
Inflation	Herd	0.024	

a *https://vmrd.com, accessed 2016, and anecdotal accounts of the diagnostic testing process*.

b *Disaggregated farm expenses found at: https://www.agmanager.info/kfma/ Costs with value ranges provided were estimated assuming a gamma distribution and rates with value ranges were estimated assuming a beta distribution*.

c *Author calculations*.

We next re-estimate the post-infection costs relying on morbidity and mortality rates document in the literature (4, 5, 20) with updated market prices from December 2016. The expected costs include death loss, culling, abortion, and private control costs. All costs are reported in USD. The costs of death loss are evaluated at the cost for a cow ($1,500) and a bull ($2,500) at the death rate of 32.5% for cows and 3.5% for bulls (5). Culling costs of $285 for cows and $1560 for bulls account for weight loss (21%) and lost revenue from lower market prices (reduction of $0.17 per lb). We assume a 1,400-pound cow and 2,000-pound bull with a cull rate of 6.4% each (20). Abortion costs are based on 2016 market prices for calf heifers ($1.20/lb) and steers ($1.30/lb), multiplied by the expected loss in pounds for each (550 and 500, respectively) at an abortion rate of 6.7%. We estimate the remaining animals receive treatment at $150 per head, which is the average cost of treating the entire herd, including labor costs, dispensing fees, and examining the animals (4, 20). We exclude production losses due to weight loss in chronic cases as cows tend to recover the full weight within a year (20). Following Alderink and Dietrick (5), we estimate a herd composition of 96% cows and 4% bulls.

### Treatment Costs

The costs of treatment options likewise appear in Table 1. Chlortetracycline feed additives ($13.80/head) and antibiotic injections ($37.40/head) are calculated using costs for veterinary services obtained from published sources from suppliers, soliciting information from practicing veterinarians, and validated against extant literature (25). The vaccination costs capture the range of current market prices for selected anaplasmosis vaccines, at $11.70/head. We adopt a constant farm labor cost of $17/h/herd (25), which is reflected in the parameter values for each treatment strategy. We calculate the diagnostic testing costs based on the cost per test, including the cost for drawing blood at $0.50/head. Each treatment cost is discounted at 4% annually. For cattle introduced into the herd, we do not assume additional costs to handling the animal, as the outside animal is being transported to the premise, and presumably passes through a herd health protocol.

### Decision Uncertainties

The improved specificity (99.7%) and sensitivity (100%) of the current recommended test (15) suggests the likelihood of herd infection instead of the accuracy of the test represents a primary source of decision uncertainty. These conditions lead us to define infection uncertainty in two ways. First, we assume a perfect diagnostic test by using a binary test outcome, where testing with treatment results in the presence of anaplasmosis with a probability of one or absence with probability of zero. Next, each anaplasmosis control decision is conditional on the initial state of the production system, subject to a random probability of infection and base level of seropositivity, ranging from 0 to 100%. If the producer is in a disease-free region, wherein the system has no seropositive cases, the producer then faces a random probability of infection. If the producer resides in an endemic region, seropositive cases likely exist in the herd, and the producer faces a random probability of infection. Buffer regions are less clear. Both observed initial states and the range of infection probabilities characterize a non-endemic buffer region. Once infected, with no preventative treatment during the production season, we assume animals stay infected and recover or are removed from the herd. Removed animals are replaced at current market prices. Replacement animals also represent a source of infection. Otherwise, once tested and treated, we assume animals remain uninfected during the production season. For simplicity, and to focus on the diagnostic testing strategy, we assume preventative treatments are 100% effective.

### Analytical Strategy

**Figure 1:**
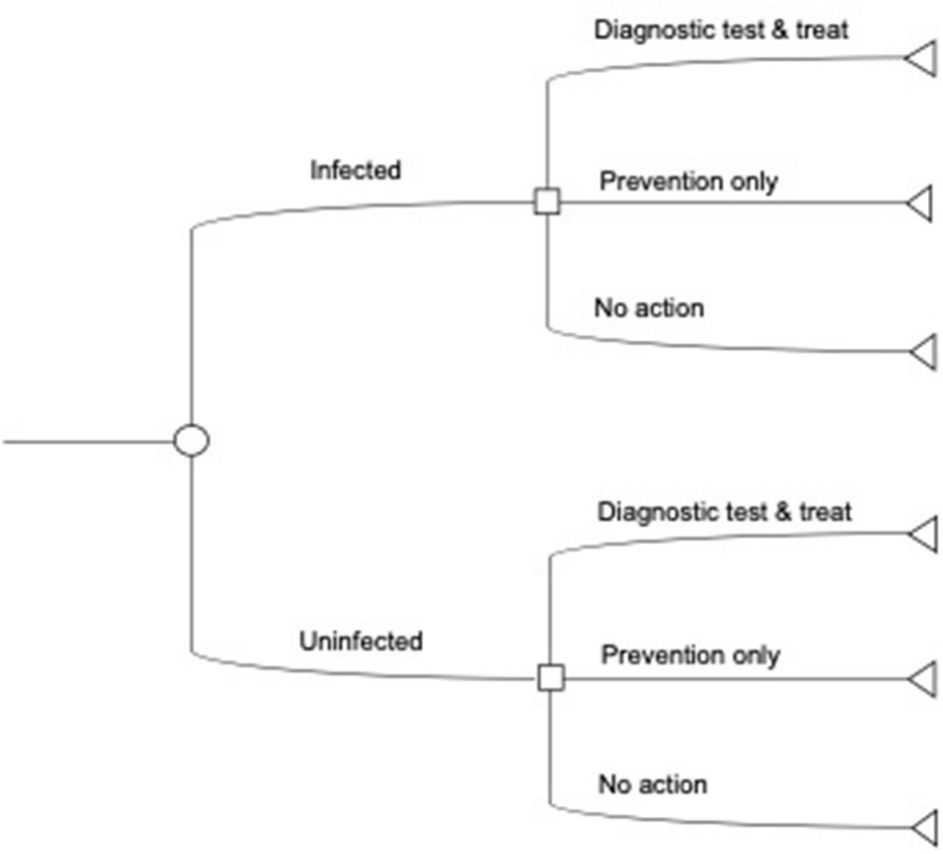
Illustrative decision tree for diagnostic tests. The producer first observes the initial infection state in the herd. Next, the producer decides to use a diagnostic test with feed or preventative treatment, only apply preventative treatments, or take no action and incur post-infection costs and losses. The decision tree here does not show the costs associated with each decision, the probability of infection, or the decision over multiple time periods (see Supplementary Materials).

The objective of our analysis is to identify the minimal cost anaplasmosis herd management strategy with diagnostic testing, given a random, exogenously determined, probability of herd infection. The economic decision model, controls, and treatments from above guide our conceptualization of the anaplasmosis control decision such that a producer minimizes losses to anaplasmosis under a finite stage, stochastic dynamic decision process (26). Figure 1 outlines the control decision at one time point, with additional details on the modeling process in the Supplementary Material (Analytical strategy).

Our model follows a 2-year planning horizon to be consistent with our economic decision model and recommended control strategies. We assume an initial base level of seropositive animals in the herd. Given the current state of new infections in the herd, the producer decides to use a diagnostic test with treatment, preventative treatment only, or takes no preventative action but follows up with post-infection treatment. We consider six total control measures: two preventative control measures (chlortetracycline feed additives to the entire herd every year; vaccinate the entire herd every year); three diagnostic testing controls (testing with vaccination; testing with feed additives; testing with post-infection treatment control); and post-infection treatment control only. The diagnostic testing with chlortetracycline feed option includes testing to identify the seropositive cattle and feed additives in each year. Diagnostic testing with vaccination involves testing in the 1st year with vaccination but no testing in the 2nd year (22). Across testing strategies, testing identifies the seronegative animals to treat, as opposed to treating the entire herd. The testing decision and a random probability of infection determine the subsequent infectious state and control strategies. Backwards iteration calculates a sequence of optimal decisions based on the costs of associated losses at each decision point and disease state. The calculation of the final expected net costs collapses into a single assessment that balances the current cost of diagnostic testing and treatment with future cost morbidity and mortality, conditional on infection state and the probability of infection.

We estimate the decision model under the assumption of a 100 head herd. The average herd size in the United States is around 40 animals but herds of 100 animals and greater account for the majority of the national beef-cow inventory (27). We compare these results to infection when one animal is introduced into the herd to demonstrate the additive cost effect within plausible ranges of future anaplasmosis morbidity and mortality costs (5, 21). To augment the outcomes from the model and provide direction for future studies to incorporate disease transmission parameters, we additionally frame the results within the context of the three different infection regions: endemic region (100% infected), disease free region (0% infection), and non-endemic buffer region (positive but not 100% infected).

### Sensitivity and Robustness Analyses

To further examine sensitivity and robustness of results, we incorporate uncertainty into the cost parameters by varying the proportion of death losses and treatment costs. Resampling from the distribution of losses and costs retrieves a range of estimates with confidence intervals to use in the decision model. We then assess the robustness of our expected costs by comparing to the expected costs from previous anaplasmosis outbreaks, adjusting for inflation. The uncertainty analyses and robustness checks of our parameters are discussed within the presentation of the primary results.

## Results

### Expected Costs and Losses

We calculate the expected economic cost of anaplasmosis at $660/head (CI: 621–699) (Table 2). This is the clinical cost of infection, or the expected cost conditional that the animal is infected with certainty. Of this, death loss contributes to the majority of anaplasmosis economic losses at 66%, followed by 16% from treatment, 11% from chronic cases, and 7% abortions. Incorporating the range of mortality rates and treatment costs into the estimation shows death loss likely ranges between 63 and 69% and treatment costs between 15 and 18%, at a 95% confidence level. With a death loss of 10%, the cost decreases to $285 (CI: 263–309). For comparison, directly adjusting the costs from the literature for inflation, in 2016 dollars, the average clinical cost of anaplasmosis per head of cattle is $793/head. The elevated costs result in part from including weight loss of chronic cases that completely recover for a total cost breakdown of 54% from death loss, 14% from weight loss, 8% to chronic cases, 9% abortions, and 15% treatment. Relative price changes in the market over time and differences in calculation methods account for the remaining percentage differences (5). A visual relationship between the expected average clinical cost of future morbidity and mortality, with variation in treatment costs and mortality rates, appears in the Supplementary Figure 2 (Sensitivity Analyses).

**Table 2:** Estimated average costs of anaplasmosis by estimation method and production impact.

	**Percent of average cost (%)**					**Average cost ($/head in USD)**
	**Death loss**	**Chronic cases**	**Abortions**	**Treatments**	**Weight loss**	
**Estimation method**						
Minimal death loss	10	28	16	46		285
Expected death loss	66	11	7	16		660
Inflation adjusted^*^	54	8	9	15	14	793

* *Retrieved from (5)*.

### Decision Analysis With Uncertainty

Under the assumed costs and model structure, the optimal policy is to test in year 1 and to vaccinate seronegative cattle as needed for years 1 and 2. Select outcomes of the dynamic programming model appear in Supplementary Table 1. In general, diagnostic testing coupled with a preventative control option reduces the costs of control relative to preventative only control measures. Because diagnostic testing allows for the identification of seronegative animals, a producer does not need to apply unnecessary treatments. Stated differently, as the number of seronegative cattle in a herd increases, the opportunity cost of not using diagnostic testing increases.

In our framework, it is never optimal to preventatively feed additives to the entire herd in perpetuity nor to preventatively vaccinate the entire herd in perpetuity. For insight, Table 3 shows the cost comparison between treating one animal and the entire herd for the separate control strategies. Preventing infection in the entire herd of 100 animals with vaccination on average costs $1,120 (CI: 1,020–1,220) and $1,400/herd (CI: 1,290–1,500) for feed additives. Here, the producer experiences cost inefficiencies over time by treating already infected and recovered cattle. Likewise, using no preventative control measures, and only applying post-infection treatment as a herd health management strategy can be costly and risky.

**Table 3:** Estimated costs for anaplasmosis control strategies by herd size (in USD).

	**Diagnostic testing**	**Preventative vaccination**	**Preventative feed additive**	**Post-infection antibiotic injections**
$ per 1 cow	7	11.7	13.9	35.1
		(10.2–12.2)	(12.9–15.0)	(33–37.3)
$ per 100 cows	700	1,120	1,400	3,890
		(1,020–1,220)	(1,290–1,500)	(3,300–3,700)

Several important caveats deserver further explanation. It is almost never optimal to ignore preventative control measures as a herd health management strategy. Nevertheless, over our specified control strategies, post-infection treatment is likely the practical response in a disease-free region with no underlying seropositive cases, and when a producer experiences an exogenous unexpected random infection. A higher risk of anaplasmosis exists in herds with an initial, higher proportion of seronegative cattle, while herds with an initial lower proportion of seronegative cattle remain at lower risk to anaplasmosis. In the case of higher proportions of seronegative cattle in a herd, then relative to the optimal control, other diagnostic testing controls and preventative controls become more competitive in terms of costs. This is because a larger number of cattle in the herd need to be protected from the risk of infection. In contrast, for herds with a smaller proportion of seronegative cattle, such as in an endemic region, diagnostic testing controls are substantially less costly than preventative controls. Finally, as the exogenous random probability of infection increases, a producer risks higher costs and losses across the two time periods. Without preventative control, producers risk output losses and asset losses from morbidity and mortality, as well as the opportunity cost of replacing cattle removed from the herd. Those under the post-treatment only control realize higher exposure to infection and, subsequently, higher losses and costs.

### Additional Applications

A producer may also consider the risk of introducing one animal into the herd. In endemic regions with a higher proportion of seropositive levels in a herd and for low levels of infection in a buffer region, a producer should weigh the cost of the diagnostic test at $7.00/head with the cost of preventative treatment through either feed additives or vaccination, against the expected cost of a clinical case $660/head (Table 3). In a disease-free or buffer region where a higher proportion of seronegative cattle face a low probability of infection, the producer decision involves whether to test the animal for anaplasmosis but weighs the decision against the expected costs of infection in the herd. Testing with preventative treatment ranges from $18-$22/head depending on preventative treatment strategy. The producer incurs no cost if the animal is not a carrier. If the animal is a carrier and infection occurs, the producer can expect future morbidity and mortality costs in the herd.

## Discussion

Increasing evidence on the prevalence of bovine anaplasmosis in the United States, coupled with the availability of early detection and prevention measures, prioritizes the need for updated cost estimates on the disease burden. Our study finds an expected cost for a clinical case of anaplasmosis in the United States of $660/head. Adjusting the previous estimate of $424/head for inflation reveals a cost of $793/head, which reflects an upper bound estimate that removes the potential for infected animals to regain lost weight (5). Reducing death loss cuts the costs to $285/head. In practice, shifting the cost burden to treatments and/or testing may require additional resources over time through labor and equipment. However, when applied early, these strategies can avoid production losses due to death loss or weight loss when infection is certain. Consequently, applying preventative measures can have a large impact on reducing the producer's burden of anaplasmosis.

Diagnostic testing helps reduce a producer's uncertainty toward efficient disease control by providing improved information on the herd infection level and the number of potential infections. Testing does not change the probability of infection in-of-itself but rather provides additional disease management options for the producer. With testing, producers may then reduce opportunity costs from late and inefficient treatment (28). Only in the disease risk extremes, such as in a disease-free region or endemic region, do cost tradeoffs exist between diagnostic testing, prevention only, or post-infection treatment. In these regions, a producer may possess sufficient a priori information on seroprevalence to efficiently treat and protect the entire herd against future infections (29, 30). Otherwise, the producer may also choose to separate susceptible animals to reduce infection risk or randomly test a sufficient number of animals to detect herd seropositivity levels (31).

The relative cost of control options may likewise affect the optimal control strategies for anaplasmosis. The current cost assumptions suggest diagnostic testing with feed additives is sub-optimal to diagnostic testing and vaccination. However, an increase in vaccination costs may make feed additives more appealing relative to vaccination. Currently, vaccine availability is limited in the United States (32), with a few experimental vaccines in progress (22), which could increase vaccine costs from a higher price per dose or if less effective vaccines require supplemental control measures. Similarly, recent federal laws to restrict the application of feed additives to use by or on the order of a licensed veterinarian likely increase the real costs of feed additives (33), but the relative ease and familiarity of feed additives suggests some producers may find the simplicity of this control alternative appealing (21). Shifting the demand to vaccination from the familiar feed additives will likely require additional cost disincentives, such as taxes or other price penalties that match the marginal gain of antibiotics (34). The magnitude of the incentives to shift demand requires further investigation into the real cost of disease management strategies in the United States, including eliciting producer preferences for the three treatments, examining if social motives influence control decisions, and the impact of regulations on the use of antibiotics.

Additional benefits from testing likely accrue over time. Producer's may reduce costs by combining with treatment to spread the fixed cost component of veterinary fees and travel across more animals. For example, if a large proportion of a herd is affected by a disease, and if fixed costs are low, producers may be more likely to treat animals despite the relatively large total costs (35). Likewise, the increased presence of blood tests in cattle (e.g., for pregnancy), suggests that cost savings may occur when herd health tests are combined. Importantly, over time, producers may experience higher revenues from diagnostic testing. Optimizing the revenue for diagnostic testing could lead to heavier weights for healthier animals relative to sick animals (36), fewer abortions (37), higher prices attributed to reputation (38), or free-from disease status in trade (39).

The results of our study offer preliminary guidance on incorporating diagnostic testing into anaplasmosis control strategies and update our existing estimates on the cost of anaplasmosis in the United States. Relying on empirical evidence of production losses and cost estimates from anaplasmosis in multiple locations across the United States strengthens the validity of our analysis. Limited variation in the types of anaplasmosis treatments to feed additives, post-infection antibiotics, and vaccines, along with relatively stable price points for the treatment options improves the precision of our estimates. We also benefit from assessing costs for a highly accurate diagnostic test, which reduces uncertainty in the parameters and overall analysis. Yet, essential research opportunities remain to address broader issues in anaplasmosis control. This includes more rigorously quantifying alternative control measures, examining transmission rates between and within herds conditional on disease regions (i.e., endemic, disease-free, buffer), exploring infection rates over time within and across disease-free and buffer regions, and altering the model assumptions to include producer trade restrictions across regulatory environments. Agent-based models to accommodate disease transmission and producer preferences could capture these complexities to help target the provision of diagnostic testing. Incorporating market conditions through revenue gained and long-term herd health impacts would then more accurately assess the impact on long-run profitability. Finally, quantifying the aggregate impacts to regions in the United States, and in total for the United States would require a different modeling approach than the current paper and would be relevant for determining national policy and priority setting.

## Conclusions

We find that diagnostic testing for the early detection of anaplasmosis in cattle is cost minimizing in the presence of infection and with more than one susceptible animal. Diagnostic testing increases a producer's information on herd infection levels so that the producer may apply control strategies more efficiently. Within the disease and regulatory context of the United States, policy makers should carefully consider investing in diagnostic testing to increase the producer's revenue and long-run cost saving potential.

## Data Availability Statement

The original contributions generated for this study are included in the article/Supplementary Material, further inquiries can be directed to the corresponding author/s.

## Author Contributions

TM conceptualized the manuscript and supervised. AR and TM wrote, edited, conducted analyses, interpreted results, and approved the final manuscript. All authors contributed to the article and approved the submitted version.

## Conflict of Interest

The authors declare that the research was conducted in the absence of any commercial or financial relationships that could be construed as a potential conflict of interest.

## Publisher's Note

All claims expressed in this article are solely those of the authors and do not necessarily represent those of their affiliated organizations, or those of the publisher, the editors and the reviewers. Any product that may be evaluated in this article, or claim that may be made by its manufacturer, is not guaranteed or endorsed by the publisher.
