# Supplementary material for: Economic Benefits of Diagnostic Testing in Livestock: Anaplasmosis in Cattle
Source: Front Vet Sci. 2021 Aug 3;8:626420. doi: 10.3389/fvets.2021.626420 (PMC8369028; doi:10.3389/fvets.2021.626420)
Supplement: Supplementary file 1 [file Data_Sheet_1.pdf]

Supplementary Material

1 Analytical strategy

The analytical strategy follows (Conrad and Clark 1987) for a discrete, finite time, stochastic dynamic programming model. We assume there is a base level of seropositive cattle present in the herd ranging from 0 to 100%. First, given this is a dynamic programming model, the state variable indicating new infections, X_t, is observed before selecting the diagnostic testing decision, Y_t. Here, t=0,1,…,T represent the time periods with k representing the remaining time periods. For this study T=2. Next, we assume the test decision Y_t and a random effect w_t occur in period t with k remaining time periods. The stochastic equation to identify future states in the stochastic process takes the form

$$f(X_{t+1}) = f(X_t, Y_t, w_t)$$

where the state variable X_t =1 indicates the presence of new infections and X_t= 0 indicates otherwise. The diagnostic testing decision Y_t is a discrete variable of Y_t = 1 for diagnostic testing and treatment and Y_t = 0 signifies no testing but post-infection treatment. Note that there are additional options that include Y_t = 2 for preventative treatments. The random variable w_t is *iid* with binary outcomes 1 or 0, with probability of infection, γ, and with probability of no infection, $1-\gamma$.

Defining the value function $J_k(x)$ as the minimum total discounted expected costs with k periods remaining, given $X = x,\ Y = y$ and assuming a conditional probability distribution with the random effect $W = w$, the optimization problem becomes:

$$J_{k+1}(X) = \min_{Y} \left\{ C(X,Y) + rE\left\{J_k\big(f(X,Y,W)\big)\right\}\right\}$$

where r is the discount factor. Here, r=0.04 for the empirical analysis. We apply backwards iteration to retrieve the optimal policy and minimized discounted expected cost of losses, conditional on infection state and test decision.

The empirical analysis further assumes that once animals are diagnostically tested, they can be identified as seropositive or not, and managed accordingly. Once infected, with no preventative treatment during the production season, we assume animals stay infected and recover or are removed from the herd. Likewise, once tested and treated, we assume animals remain uninfected during the production season. Animals removed from the herd are replaced at current market prices. Terminal values for each state are the discounted present value in perpetuity for the specific control.

The option to diagnostic test and vaccinate is the dominate and optimal policy across parameter values. Table S1 provides outcomes of the model for selected parameters across the control options. These values reflect costs in years 1 and 2, as well as costs at the terminal values. High levels of seronegative herds have larger number of cattle at risk and, consequently, higher control costs. Higher random infection rates increased the cost of no preventative actions with only post-infection treatment.

Table S1 Outcomes of the dynamic programming model for selected parameters.

	Seronegative Level in Herd			Random Infection
	0.9	0.5	0.1	
**Control**	Total $	Total $	Total $	
No Preventative Action, Post-Infection Treatment	$47,512	$45,840	$44,168	0.7
No Preventative Action, Post-Infection Treatment	$32,646	$26,470	$20,294	0.3
Preventative Chlortetracycline Feed Additive Entire Herd	$37,260	$37,260	$37,260	0.3
Preventative Vaccination Entire Herd	$31,509	$31,509	$31,509	0.3
Diagnostic Test and Antibiotic Treatment	$92,282	$51,890	$11,498	0.3
Diagnostic Test and Vaccination	$29,058	$16,455	$3,851	0.3
Diagnostic Test and Chlortetracycline Feed Additive	$34,934	$20,030	$5,126	0.3

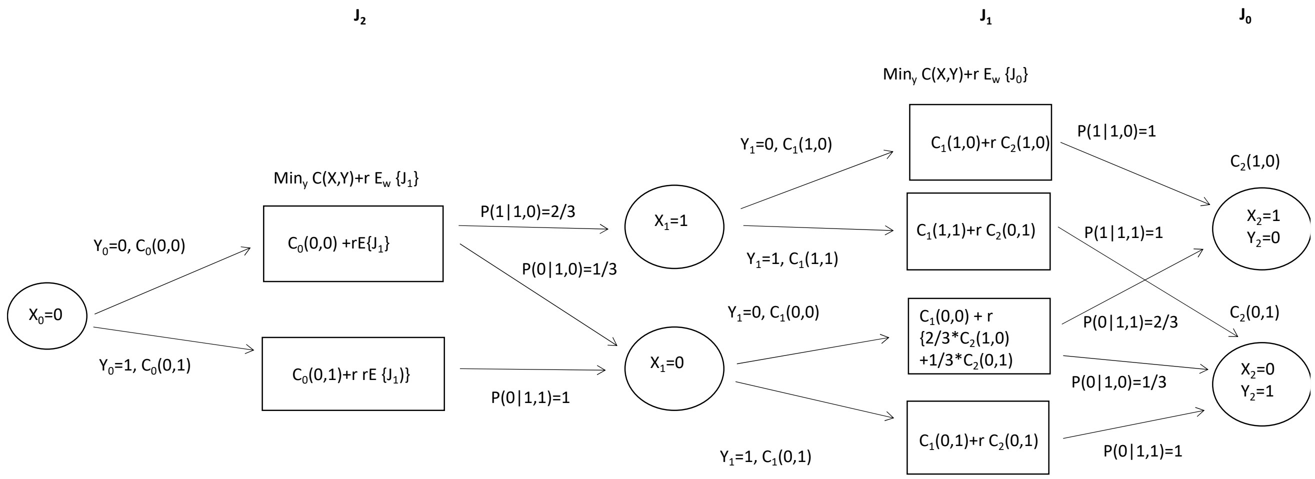

Figure S1 shows an illustrative diagram for t=0,1,2 periods to determine optimal testing policies between controls Y=0 and Y=1. The initial state is X_0 =0 with random probability, $\gamma = 2/3$. Once infected, we assume animals remain infected. Additional controls applied in this study have identical structure as Y=1 above. Likewise, once tested and treated or treated with preventative treatments, we assume animals remain uninfected during that period.

2 Sensitivity Analysis

For each expected cost analysis, a gamma distribution was used to assess the confidence in parameter estimates. For the mortality rates, a beta distribution was assumed. Data to determine the distributions came from expert opinion and was cross referenced with extant literature.

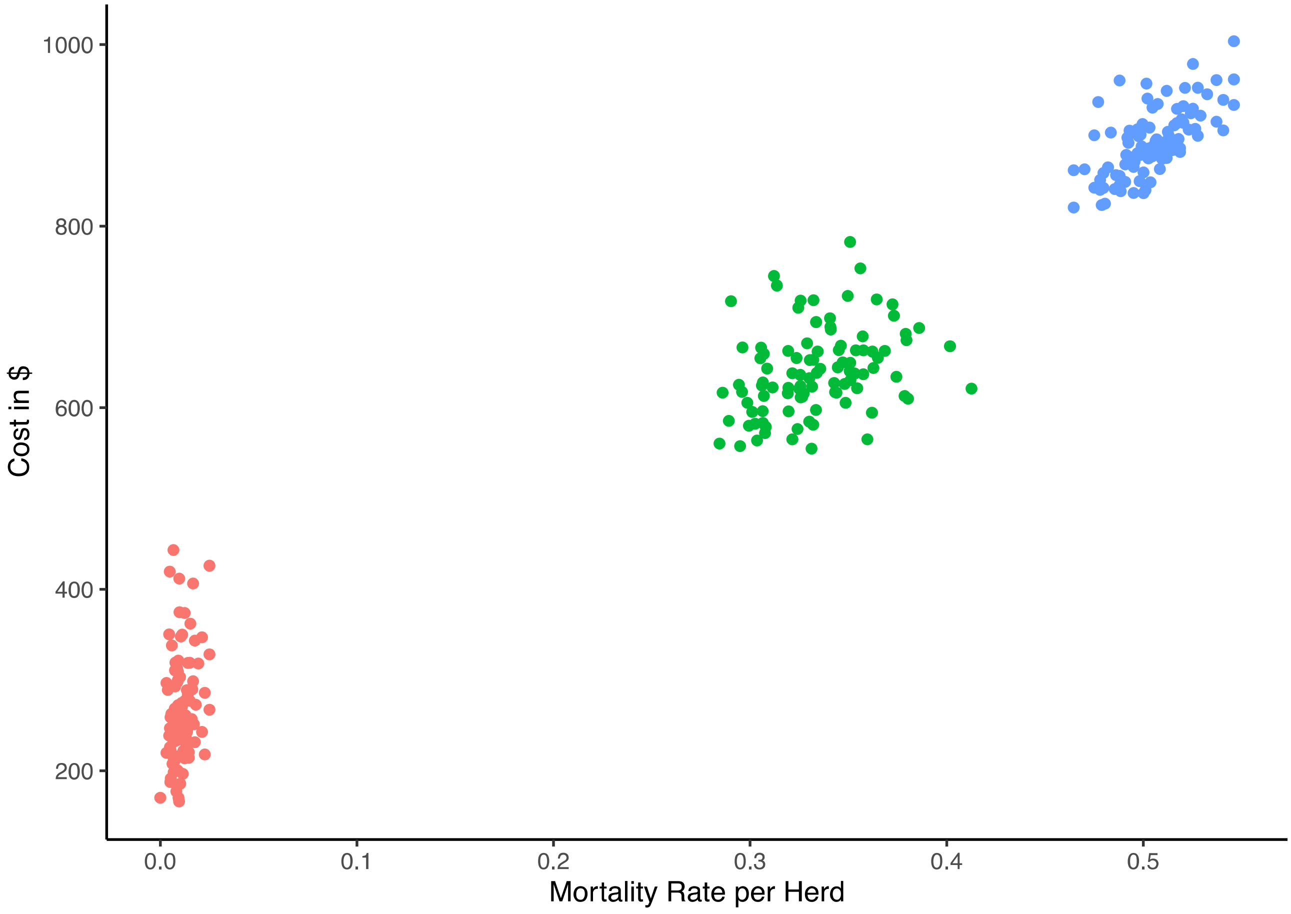

Figure S2 Relationship between the expected average cost of anaplasmosis costs and losses with expected mortality rates. Variation in treatment costs is included in the calculation.

References

Conrad, Jon M., and Colin Whitcomb Clark. 1987. *Natural Resource Economics*. Cambridge University Press.
